# Development of Sustainable Polymer Composites Containing Waste Glass and Natural Fibers for Strengthening Purposes

**DOI:** 10.3390/polym17081116

**Published:** 2025-04-20

**Authors:** Cihan Karademir, Hasan Murat Tanarslan, Çağlar Yalçınkaya, Mustafa Furkan Güler, Hasan Ateş, Kutlay Sever, Yasemin Seki, Metehan Atagür

**Affiliations:** 1 The Graduate School of Natural and Applied Sciences, Dokuz Eylül University, İzmir 35390, Türkiye; cihan.karademir@kavram.edu.tr (C.K.); m.furkan.guler@adu.edu.tr (M.F.G.); 2 Section of Construction Technology, İzmir Kavram Vocational School, İzmir 35230, Türkiye; 3 Department of Civil Engineering, Faculty of Engineering, Dokuz Eylül University, İzmir 35390, Türkiye; murat.tanarslan@deu.edu.tr; 4 Department of Civil Engineering, Faculty of Engineering, Aydın Adnan Menderes University, Aydın 09010, Türkiye; 5 Environmental Health Program, İzmir Kavram Vocational School, İzmir 35230, Türkiye; hasan.ates@kavram.edu.tr; 6 Department of Mechanical Engineering, İzmir Katib Çelebi University, İzmir 35620, Türkiye; kutlay.sever@ikcu.edu.tr; 7 Department of Textile Engineering, Faculty of Engineering, Dokuz Eylül University, İzmir 35390, Türkiye; yasemin.seki@deu.edu.tr; 8 Department of Metallurgical and Materials Engineering, İzmir Katib Çelebi University, İzmir 35620, Türkiye; metehan.atagur@ikcu.edu.tr

**Keywords:** hybrid composites, flax fiber, hemp fiber, glass fiber, mechanical properties, thermal properties

## Abstract

This study investigates the development of sustainable polymer composites for structural strengthening by incorporating waste glass fibers and natural fibers (flax and hemp) into an epoxy matrix, in response to the growing environmental concerns. Mechanical, thermal, and durability-related properties were evaluated through tensile testing, dynamic mechanical analysis (DMA), thermogravimetric analysis (TGA), water absorption, and water immersion aging tests. Results showed that incorporating waste glass fibers enhanced the tensile strength and thermal decomposition temperature by 88% and 5.4%, respectively, compared to composites reinforced with solely natural fibers. Water absorption tests indicated that waste glass fiber-reinforced hybrid composites exhibited lower water uptake than flax and hemp fiber-reinforced composites. After water immersion, the tensile strength loss was recorded as 22, 25, and 8.5% for the composites reinforced with hemp, flax, and waste glass fiber, respectively. The findings confirm that incorporating waste glass fibers into natural fiber composites effectively mitigates moisture sensitivity and improves mechanical performance. Hybridizing flax and hemp fibers with waste glass fibers provides a practical and sustainable approach to enhancing composite performance, making them a viable alternative for strengthening reinforced concrete structures requiring long-term resistance. The recycled waste glass fibers employed in this study offered comparable mechanical performance while drastically lowering raw material consumption and environmental impact, in contrast to virgin glass fibers frequently used in earlier investigations. This demonstrates how recycling-oriented composite design can provide both sustainability and performance benefits.

## 1. Introduction

The increasing demand for synthetic fiber-reinforced polymer (FRP) materials is frequently preferred for structural strengthening applications due to their high strength-to-weight ratios and mechanical superiority [1]. Externally bonded FRPs enhance the load-bearing capacity of reinforced concrete elements; however, the weak bond between FRP and concrete often limits the full utilization of the material’s tensile strength [2]. This limitation can cause premature debonding from concrete surfaces, resulting in brittle failure and wasting the material’s potential. Although anchoring techniques can mitigate these issues, they involve additional labor costs and potential localized weaknesses due to drilled holes [3,4].

Among synthetic fiber-reinforced polymers, glass fiber-reinforced polymer (GFRP) composites are widely used in structural engineering thanks to their mechanical properties, corrosion resistance, and ease of application [5,6]. However, the production processes of these materials involve several energy-intensive stages, including the melting of glass fibers and the curing of thermoset resins. For instance, glass fiber production requires approximately 13 MJ/kg to 32 MJ/kg of energy. At the same time, the manufacturing processes of epoxy and polyester resins consume about 76–80 MJ/kg and 63–78 MJ/kg, respectively [7]. Moreover, a considerable amount of waste and byproducts are generated during the GFRP production process, with approximately 10–15% of the total glass fiber input becoming waste during production [8]. Inevitably, used GFRP materials will become waste throughout their product life cycle. The non-biodegradability of these wastes leads to ecosystem pollution, disrupts the ecological balance, and threatens sustainability [9,10]. Given the increasing concerns over GFRP disposal, various end-of-life management strategies have been explored, including landfilling, incineration, mechanical grinding, and use in cement kilns [11,12,13]. However, each method presents environmental drawbacks, such as air pollution, energy consumption, and secondary waste generation. As an alternative, recycling approaches have been developed to recover valuable fibers and enhance sustainability. While mechanical recycling is cost-effective, it significantly reduces fiber strength. On the other hand, chemical recycling involves high energy consumption and the release of hazardous emissions, further exacerbating environmental concerns [8,14].

To address the environmental concerns associated with synthetic fiber-reinforced polymers, researchers have explored natural fiber-reinforced polymer (NFRP) composites as an alternative. Cellulose-based natural fibers such as jute, flax, and hemp offer a more sustainable and eco-friendly option due to their biodegradability and lower environmental footprint [15]. Compared to synthetic fibers, these materials present significant advantages in green material design, providing a cost-effective and carbon-emission-reducing alternative [16,17,18]. Consequently, NFRP composites are increasingly being investigated for automotive, construction, and shipbuilding applications [19,20]. Despite their sustainability benefits, NFRP composites exhibit several limitations that hinder their widespread adoption, particularly in structural strengthening applications. Their tensile strength and stiffness are generally lower than those of synthetic fiber-reinforced ones, which restricts their mechanical performance in load-bearing applications [15]. Also, natural fibers suffer from high moisture absorption, low thermal stability, and biodegradability, which can negatively affect their long-term durability [21,22]. Their hydrophilic nature leads to significant moisture uptake, degrading the mechanical and thermal properties of the composite material. Moreover, their low thermal stability causes degradation between 305 and 370 °C, making them unsuitable for high-temperature applications [23]. Various strategies have been explored to enhance the durability and performance of NFRP composites, including surface modifications, hybrid composite systems, and specialized resin–matrix combinations [24]. These modifications improve natural fiber composites’ mechanical properties and environmental resistance, making them more favorable alternatives to synthetic fiber composites. As research continues to address these challenges, NFRP materials hold promise as sustainable reinforcements, balancing environmental responsibility with engineering performance [25,26,27].

The hybrid approach has been adopted to address the challenges associated with natural fibers and develop structural strengthening polymer composites capable of fully utilizing their mechanical potential. Hybrid composites, which combine natural and synthetic fibers, enhance mechanical performance while promoting environmental sustainability. These systems reduce the brittle failure tendency of synthetic fibers and distribute stresses more evenly, enabling the development of reinforcement materials compatible with concrete and capable of fully utilizing their strength [15,28].

Incorporating natural fibers with glass fibers enhances mechanical strength, energy absorption capacity, and durability while promoting renewable resource use [29,30]. For instance, reinforcing flax fibers with specific proportions of waste glass fiber has been shown to improve the flexural strength of composites [31]. Similarly, hybridizing hemp fibers with synthetic fibers leads to notable improvements in tensile strength, stiffness, and flexural performance. In particular, functionally graded and sandwich hybrid configurations optimize the stacking sequence, enhancing the overall structural integrity [32,33].

Unlike many other researchers who use virgin or commercial-grade glass fibers in hybrid composites, this work focuses on recycling waste glass fibers, a byproduct commonly discarded during manufacture or from end-of-life GFRP components, as a reinforcement material. Previous research has extensively investigated hybrid composites composed of natural and synthetic fibers [34,35,36], but few have examined the effects of incorporating recycled glass fibers into such systems. In this perspective, the current study makes a significant contribution by looking into how the reuse of discarded glass fibers might provide equivalent or even superior mechanical performance while simultaneously providing increased environmental benefits. Recycled fibers, when compared to virgin glass fibers, reduce composites’ embodied energy and carbon footprint while also supporting circular economy objectives [37,38]. Furthermore, research such as that conducted by Fiore et al. and Sathishkumar et al. predominantly examines virgin glass fibers within hybridization strategies, while our study explores the performance and sustainability advancements possible through the substitution of these fibers with post-industrial or post-consumer waste fibers [39,40]. The difference not only addresses the issue of GFRP waste disposal but also illustrates the viability of eco-efficient high-performance composites for structural strengthening applications.

The advancement of biodegradable or recycled composite materials is essential for reducing environmental pollution, offering economic advantages, and integrating eco-friendly materials into industrial applications [41,42,43]. This experimental study aims to develop sustainable FRP composites for strengthening applications in structural engineering. Hybrid composites were produced using waste glass fibers and fabrics made of natural fibers (flax and hemp), and their potential for structural strengthening applications was discussed. Comprehensive material characterization tests were conducted to determine the developed composites’ mechanical, thermal, and environmental resistance and shed light on their performance under service conditions.

## 2. Materials and Methods

### 2.1. Materials

**Figure 1:**
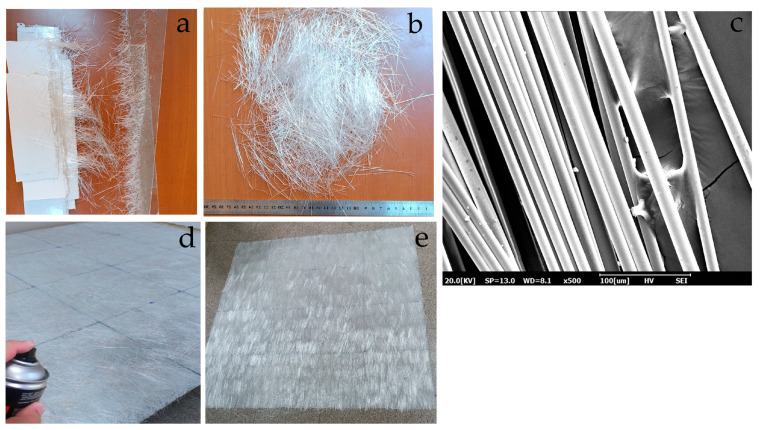
Composite waste processing steps. (**a**) Composite waste; (**b**) waste glass fibers; (**c**) SEM images of the waste glass fibers; (**d**) applying spray adhesive; (**e**) layered waste glass fibers.

In this study, waste glass fibers and two types of woven fabrics consisting of 100% flax and 100% hemp fibers were used as reinforcement materials in composite production. Glass fibers are generated as waste during the forming and resin impregnation stages of composite manufacturing processes, followed by the removal of excess material that has not been exposed to the resin (Figure 1a). The waste fibers used in this study are of the E-glass-type chopped fibers ranging from 2 to 6 cm in length (Figure 1b). The waste glass fibers were sourced from the production residues of a local composites manufacturer and were not subjected to any additional cleaning or surface treatment. The scanning electron microscopy (SEM) image showed that the waste fibers contained no visible contaminants or surface impurities, thus confirming their direct applicability (Figure 1c).

The waste fibers have a tensile strength of 1950 MPa, a Young’s modulus of 72 GPa, a shear modulus of 30 GPa, and a Poisson’s ratio of 0.21. During composite production, these waste fibers were carefully aligned as unidirectional layers to prevent fiber dispersion and control their irregular structure. A layer with an approximate areal weight of 200 g/m^2^ was formed, and a spray adhesive was applied during this process to maintain fiber alignment. As shown in Figure 1d, the spray adhesive effectively held the waste glass fibers together. The low wetting property of the spray adhesive did not hinder the penetration of the matrix resin into the fibers, ensuring proper impregnation, as illustrated in Figure 1e. The absence of any additional washing or chemical treatment on the waste glass fibers has prevented secondary waste generation and minimized resource consumption. The layering of waste glass fibers allows for their use in polymer composites, reducing environmental impacts and providing economic advantages.

**Figure 2:**
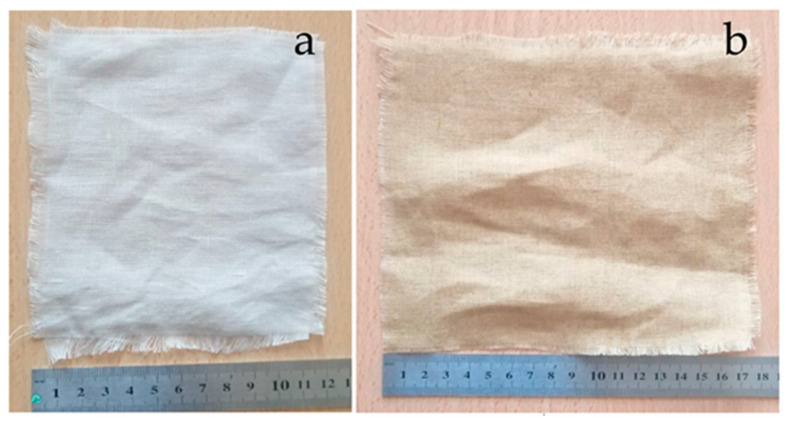
Flax and hemp fabrics. (**a**) Flax fabric; (**b**) hemp fabric.

As a natural form of fiber reinforcement, 100% flax and 100% hemp fabrics were used (Figure 2). Before polymer composite production, no chemical modification was applied to these fabrics. Table 1 summarizes the fundamental properties of these commercially available fabrics and the layered waste glass fibers.

**Figure 3:**
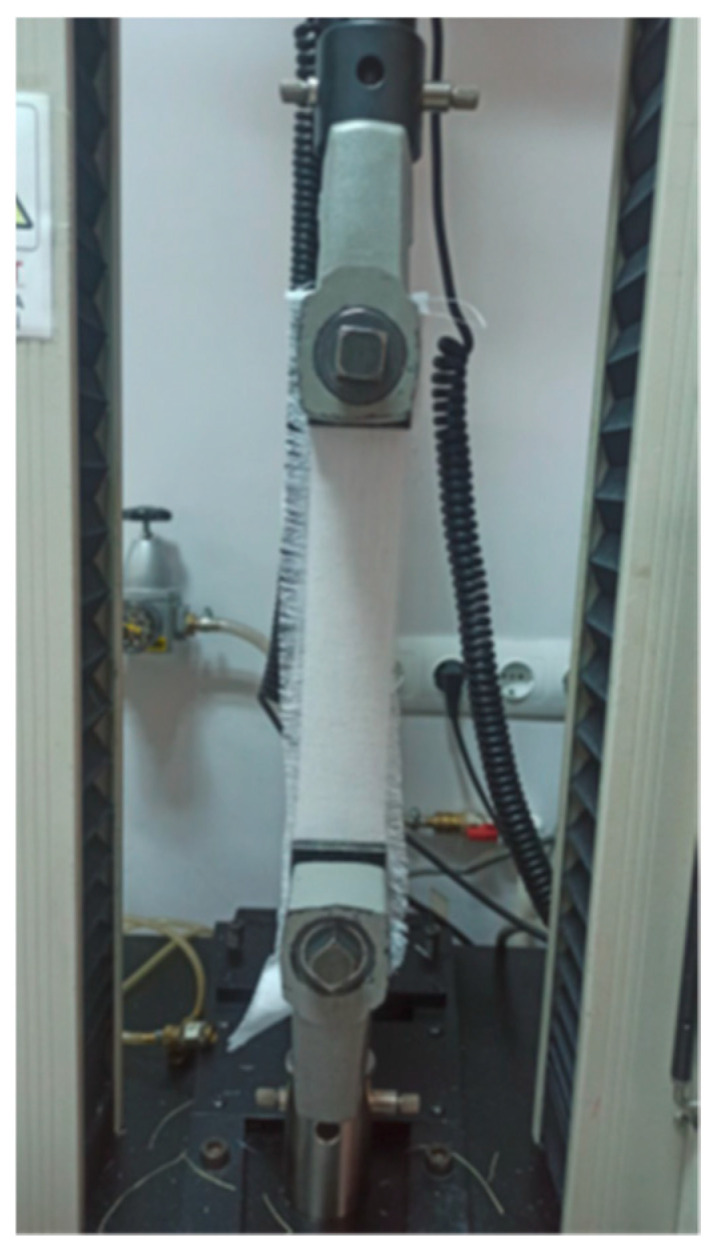
Tensile test of flax fabric sample.

The tensile strength tests of hemp and flax fabrics were conducted using the strip method specified in TS EN ISO 13934-1 (Textiles—Tensile properties of fabrics—Part 1: Determination of maximum force and elongation at maximum force using the strip method) [44]. Five specimens measuring 350 mm × 50 mm were prepared for each fabric type in both warp and weft directions. The tensile strength tests were performed at a constant rate of 100 mm/min (Figure 3). The breaking load and elongation at break values for the fabrics were obtained. Table 2 presents the tensile strength results of hemp and flax fabrics. The test results indicate that the breaking load of hemp fabric is higher than that of flax fabric, while the elongation at break values of both fabrics is similar.

### 2.2. Preparation of Polymer Composites

**Figure 4:**
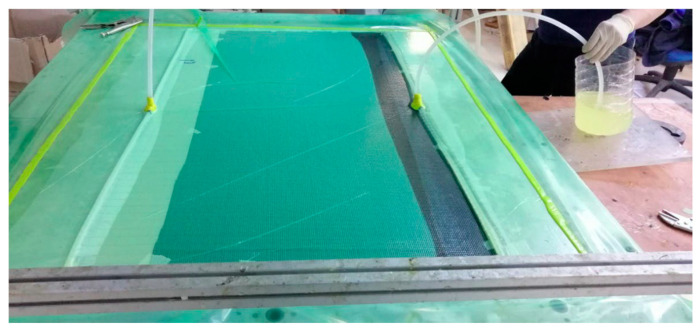
Epoxy resin impregnation by the vacuum infusion method.

There are various production methods for polymer composites. Research indicates that using epoxy resin in the vacuum infusion method minimizes the formation of air voids [45]. Therefore, the vacuum infusion method was preferred for composite production. An epoxy resin with low viscosity, high mechanical strength, and good flexibility suitable for the vacuum infusion process was used as the matrix material. The Young’s modulus and tensile strength of epoxy are 2.1 GPa and 71.4 MPa, respectively. The resin was mixed with a hardener at a 3:1 ratio and subjected to a curing process at 80 ± 2 °C for 8 h in a temperature-controlled heating table to achieve the final properties of the composites. The vacuum infusion method presented in Figure 4 allowed the epoxy resin to be uniformly distributed among the fibers under a vacuum pressure of 0.8 bar, ensuring consistent resin flow and minimizing void formation within the composite structure.

In this study, a total of five different polymer composite laminates were produced. For each reinforcement material, polymer composite laminates were prepared using six layers of waste glass fiber (G6), six layers of hemp fiber (H6), and six layers of flax fiber (F6). The hybrid polymer composite was initially designed with two layers of waste glass fibers in the middle layer and two layers of hemp fibers in the outer layers (H4G2). Subsequently, another hybrid polymer composite was developed, incorporating two layers of waste glass fibers in the middle layer and two layers of flax fibers in the outer layers (F4G2).

The layer arrangement, thickness values, and fiber content by volume of the samples obtained after the composite production process are presented in detail in Table 3. The highest natural fiber content by volume was found in the F6 laminate (47.9%), followed by the H6 laminate (42.7%). This finding indicates that hemp fiber absorbs a higher proportion of epoxy resin than flax fiber.

### 2.3. Test Methods

Tensile tests, scanning electron microscopy (SEM) analysis, thermogravimetric analysis (TGA), dynamic mechanical analysis (DMA), water absorption, and aging tests were conducted to evaluate the polymer composites’ mechanical, thermal, and durability properties.

Tensile tests were conducted following the ASTM D3039/D3039M [46] standard to determine the tensile strength, Young’s modulus, and elongation at the break of the specimens. Test specimens were prepared in dimensions of 195 × 20 mm and tested at a constant 2 mm/min loading rate. Five specimens were tested for each composite type to check for repeatability, and the length between the tensile heads (measurement length) was set to 100 mm.

A Carl Zeiss EVO 300VP (Oberkochen, Germany) SEM device was used for the observations, operating at an accelerating voltage of 2.5 kV. SEM analysis was performed to examine the fracture surface morphology of the composite specimens after tensile testing. A full cross-section sample was carefully cut from one side of the tensile fracture surfaces, avoiding damage to the naturally fractured surface, and prepared for SEM analyses. A thin layer of gold was coated onto the fractured surface using an automatic sputter coater (Quorum Q150 RES, Laughton, UK) to enhance surface conductivity and prevent charging. SEM observations on fractured surfaces were conducted at a magnification of 250 times to investigate morphological features such as fiber–matrix interactions and possible void formations.

DMA of the composite specimens was performed using a DMA Q800 dynamic mechanical analyzer in single-cantilever mode in accordance with ASTM D7028 [47]. Measurements were conducted over a temperature range from 35 °C to 150 °C, with a heating rate of 2 °C/min and a constant frequency of 1.0 Hz. TGA was conducted following ASTM E1131 [48] to determine the thermal degradation behavior and thermal stability of the polymer composite materials. The test was performed under a nitrogen atmosphere, heating the samples from 25 °C to 600 °C at a rate of 10 °C per minute while recording the weight changes as a function of temperature.

**Figure 5:**
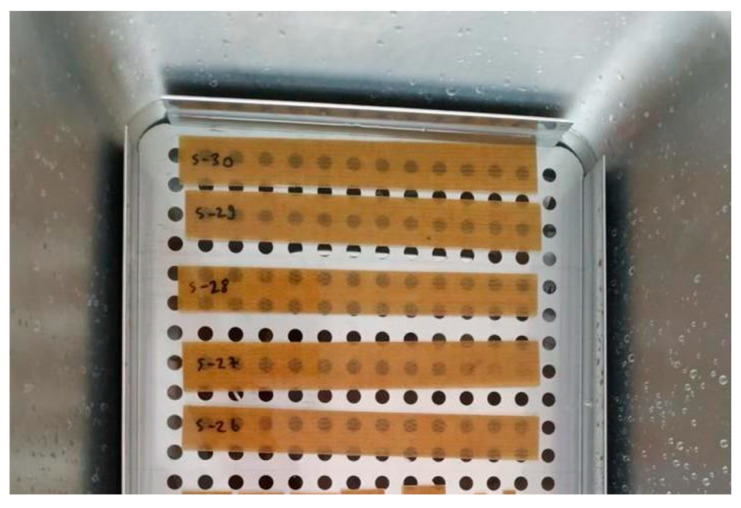
Water absorption test of the composites.

Water absorption and aging tests were carried out to evaluate the changes in the physical and mechanical properties of polymer composites when exposed to water. Specimens measuring 195 × 20 mm were subjected to water absorption testing in distilled water at room temperature (23 ± 2 °C) following ASTM D570 [49]. During the water absorption test, the specimens were removed from the water at predetermined intervals, their surfaces were gently dried with a towel to obtain saturated-surface dry conditions, and weight changes were recorded. In the aging test, the durability and long-term performance of the composite subjected to water absorption were evaluated by tensile tests performed every 5 days until the 25th day. These tests were carried out to analyze the tensile strength of the samples subjected to aging (Figure 5). The data obtained provide important information about the durability and structural integrity of composite materials in relation to environmental conditions.

## 3. Results and Discussion

### 3.1. Tensile Test Results of the Composites

The mechanical properties of hybrid polymer composites were evaluated based on sustainability and strength criteria. Tensile test results of 6F, 6H, 6G, F4G2, and H4G2 specimens are presented in Table 4. Note that Young’s modulus and tensile strength of epoxy are 2.1 GPa and 71.4 MPa in sequence. Among the composites, the 6G composite exhibited the highest tensile strength at 334.2 MPa, demonstrating that incorporating waste glass fiber enhances mechanical performance. In contrast, the natural fiber-reinforced composites, 6H and 6F, exhibited tensile strengths of 76.6 MPa and 80.7 MPa, respectively, due to the lower mechanical performance of natural fibers compared to waste glass fiber. The hybrid composites exhibited intermediate performance, with the H4G2 and F4G2 specimens achieving tensile strengths of 138.6 MPa and 149.8 MPa, respectively. These findings indicate that hybrid composites effectively integrate the environmental benefits of natural fibers with the superior strength characteristics of waste glass fibers [50].

Incorporating fiber reinforcement has enhanced the tensile properties of all composites compared to pure epoxy. In terms of Young’s modulus, the maximum value (9.4 GPa) was obtained for the 6G specimen, and this result shows that waste glass fiber considerably increases the stiffness of the composite. Meanwhile, the natural fiber-reinforced composites, 6H and 6F, exhibited Young’s modulus of 2.4 GPa and 2.2 GPa, respectively, indicating that the stiffness of natural fibers is lower due to their nature. In the hybrid composites, the elastic modulus exhibited values within an intermediate range, at 3.5 GPa for H4G2 and 3.2 GPa for F4G2. Compared to the natural fiber-reinforced specimens, these values represent an increase in stiffness but a significant decrease compared to 6G. Thus, hybrid composites exhibit greater flexibility than only glass fiber-reinforced composites while offering improved mechanical properties compared to only natural fiber-reinforced ones.

The elongation at break percentages is directly related to the ductility of the material [51]. On the other hand, because of the nature of the vacuum infusion method used for composite production in this study, the thickness of the composite specimens cannot be adjusted to a constant value. Therefore, elongation at break percentages was compared for specimens with slightly different thicknesses (between 1.8 and 2.3 mm) to provide a general insight into the ductility performance. The highest elongation at break of 10.8% was measured in sample 6F, and it was observed that flax fiber gives more ductility to the composite. In contrast, the 6G specimens with 4.6% and the H4G2 and F4G2 specimens, among the hybrid composites, exhibited elongation at a break of 4.4% and 4.8%, respectively, indicating that waste glass fiber reinforcement increases stiffness but decreases ductility. Tensile toughness determines the energy absorption capacity of the material under load. The highest tensile toughness of 40,969.5 N.mm was observed in sample 6G, and it was determined that the waste glass fiber content has a high energy absorption capacity.

The 6F specimen, containing flax fiber, exhibited superior performance compared to the 6H specimen, which includes hemp fiber, with a tensile toughness of 19,897.8 N.mm. Similarly, the hybrid use of waste glass and natural fiber further accentuates this difference. These findings indicate that flax fiber-reinforced composites have a favorable combination of high toughness and ductility. Additionally, it has been determined that glass fiber reinforcement significantly influences the rigidity and toughness properties of the composites.

**Figure 6:**
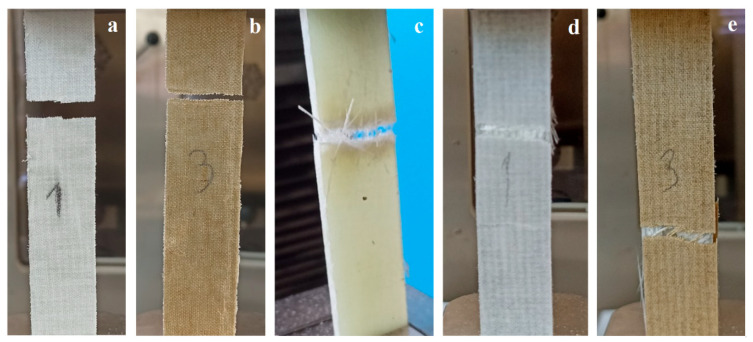
Typical fracture surfaces of the composites after the tensile test. (**a**) 6F; (**b**) 6H; (**c**) 6G; (**d**) F4G2; (**e**) H4G2.

**Figure 7:**
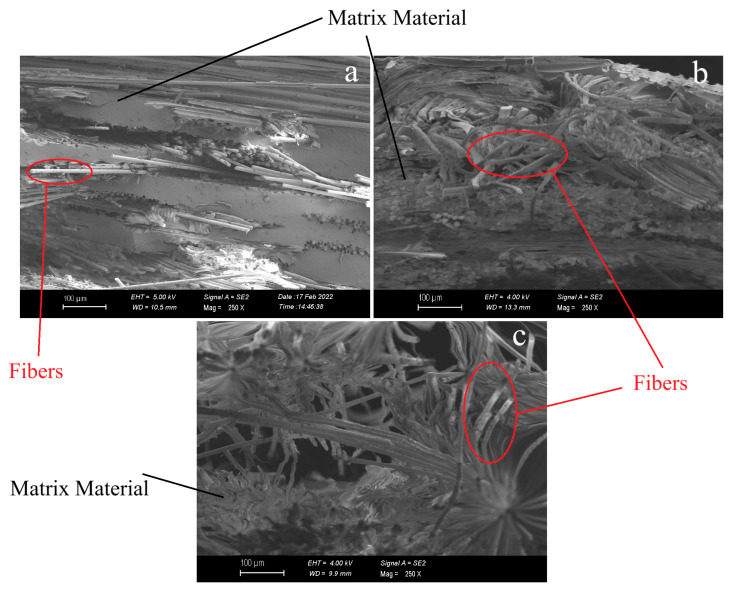
SEM images of the fracture surfaces of the composites after the tensile test. (**a**) Waste glass fiber-reinforced polymer composite; (**b**) flax fiber-reinforced composite; (**c**) hemp fiber-reinforced composite.

The typical fracture surfaces of the composites after the tensile test are presented in Figure 6. The examination of composite fracture surfaces after the tensile test revealed that no fiber pull-out was observed in natural fiber-reinforced composites (6H and 6F). The fibers fractured with the matrix in these composites, exhibiting a cohesive failure surface. However, a different failure behavior was observed in composites containing waste glass fibers (6G, H4G2, and F4G2). While some fibers ruptured under tensile loading in these composites, others detached from the matrix, resulting in fiber pull-out. The literature indicates that fiber breakage is more prevalent in cases of strong glass fiber-matrix interfacial bonding, whereas in cases of weak interfacial adhesion, fiber pull-out becomes the dominant failure mechanism [52]. Most studies report that glass fibers undergo surface modification treatments to enhance fiber-matrix adhesion, consequently increasing the tendency for fiber fracture [53,54]. In this study, the waste glass fibers were not subjected to any surface treatment but were simply oriented using a spray adhesive to form layered structures. This manufacturing approach may have hindered the optimal development of interfacial bonding, leading to fiber pull-out in certain regions during tensile testing. Scanning electron microscope (SEM) images of the fracture surfaces of polymer composites after tensile testing were obtained (Figure 7).

SEM images of waste glass fiber-reinforced, flax fabric-reinforced, and hemp fabric-reinforced composites were examined to analyze the fiber-matrix interfacial behavior. In Figure 7a, the SEM image of the waste glass fiber-reinforced composite shows that fibers, as they detach from the matrix, create voids in the polymer structure. These voids indicate the weakening effect of the method used to form the waste glass fiber layers and the spray adhesive applied during the process. Additionally, the SEM image reveals a high resin content within the composite structure. Figure 7b displays the fracture surface of the flax fabric-reinforced composite after tensile testing. SEM analysis reveals microstructural changes within the material during fracture. The irregularities on the fracture surface indicate that the material underwent deformation beyond its elastic limit under applied load, separating natural fibers from each other. In Figure 7c, the detachment of hemp fibers from the matrix is observable, indicating the material’s brittle fracture behavior. The natural structure of hemp fibers has contributed to more pronounced failure in regions with weak interfacial bonding. The fracture surface between the fibers exhibits tears and irregularities, a critical factor affecting the composite’s mechanical performance.

In this study, the mechanical properties of the developed composites were determined, and their potential use in strengthening reinforced concrete beams was evaluated. The tensile strength of the produced composites was compared with that of natural and synthetic fiber-based FRP composites reported in the literature (Table 5). According to the related literature, the tensile strength of natural fiber-based FRPs varies widely. For instance, flax fiber-based composites exhibit tensile strengths ranging from 59 to 252 MPa, and hemp fiber-based composites reach up to 156 MPa. In comparison, jute and sisal fiber-based composites display values between 17 and 136 MPa, respectively. The 6H and 6F composites developed in this study fall within these ranges, indicating limited potential for strengthening applications. Synthetic fiber-based composites, particularly those reinforced with glass fibers, achieve higher tensile strengths, ranging from 124.5 to 377.6 MPa. The 6G composite, produced solely from waste glass fibers, reaches this level, offering a sustainable alternative to synthetic fibers. Depending on the combination of natural and synthetic fibers, hybrid composites exhibit tensile strengths between 59 and 302 MPa, respectively. The H4G2 and F4G2 composites developed in this study lie within an intermediate range and are considered suitable alternatives for strengthening reinforced concrete elements. A similar trend was observed in Young’s modulus values.

Based on the results from the tensile tests, the 6H, 6F, 6G, H4G2, and F4G2 composites developed in this study provide a viable alternative for strengthening reinforced concrete beams compared to similar FRP materials in the literature. Notably, hybrid composites combine the advantages of natural and synthetic fibers, delivering a balanced solution in terms of mechanical performance and sustainability.

### 3.2. TGA of the Composites

**Figure 8:**
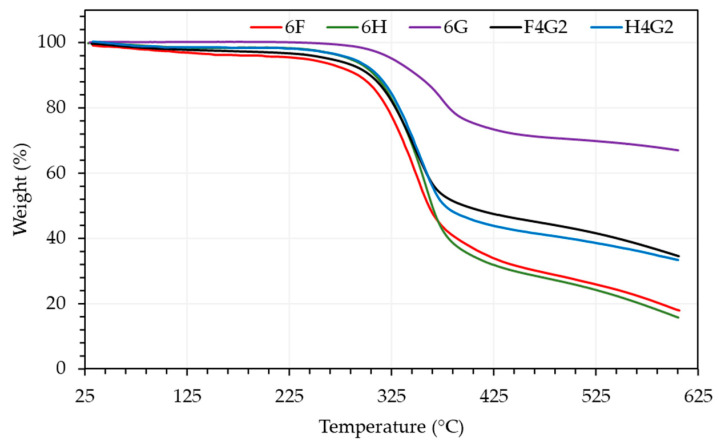
Temperature-dependent mass loss of composite samples from TGA.

TGA is a widely utilized thermal analysis technique for determining the temperature-dependent mass loss of materials. Based on TGA, Figure 8 illustrates the mass loss trends of fabricated composite samples as a function of temperature increase over the 0–600 °C.

Furthermore, Table 6 provides the initial decomposition temperatures at 5% mass loss, the maximum decomposition temperatures, and the ash content of the composites. All samples exhibited significant mass loss within the 250–400 °C range. The initial decomposition temperature of the 6H composite was determined to be 284.54 °C, surpassing that of the flax fiber-reinforced 6F sample, which exhibited an initial decomposition temperature of 237.68 °C. Regarding maximum decomposition temperatures, the 6F sample reached a peak decomposition at 348.24 °C, while the 6H sample decomposed at 350.96 °C.

Natural fibers primarily consist of cellulose, hemicellulose, and lignin, which influence their physical properties. These thermally sensitive components decompose at distinct temperature ranges [66]. Lignocellulosic materials typically degrade between 150 and 500 °C, with hemicellulose decomposing at 150–350 °C, cellulose at 275–350 °C, and lignin at 250–500 °C [67]. In the literature, the thermal decomposition of pure epoxy is reported to initiate at 294 °C [68], with a maximum decomposition temperature of 354.5 °C [69]. For the 6G sample, thermal decomposition began at 326.04 °C, with a maximum decomposition temperature of 369.98 °C. These values exceed those of pure epoxy, indicating that the incorporation of waste glass fibers into the epoxy matrix enhances the thermal stability of the composites. This effect is also observed in hybrid composites. Compared to 6H and 6F, hybrid composites demonstrate superior thermal stability. Specifically, the H4G2 sample exhibited an initial decomposition temperature of 286.57 °C and a maximum decomposition temperature of 346.15 °C, while the F4G2 sample showed an initial decomposition at 264.64 °C and reached a maximum decomposition temperature of 342.75 °C.

The total mass loss of the 6G sample was notably low at 32.98%, which can be attributed to the absence of organic fibers. The inclusion of glass fibers not only increases the decomposition temperatures of these composites but also reduces the total weight loss. The F4G2 and H4G2 samples exhibited lower mass loss than 6F and 6H. In conclusion, while natural fiber-reinforced epoxy composites and hybrid systems tend to decompose at earlier stages than pure epoxy, adding glass fiber reinforcement improves thermal stability. This enhancement is evident in the higher decomposition temperatures and reduced mass loss observed in glass fiber-reinforced samples. Numerous studies emphasize the remarkable benefits of incorporating glass fibers into natural fiber-reinforced composites, particularly in enhancing thermal stability. Braga and Magalhães reported that adding glass fibers to jute-based composites significantly reduced the overall mass loss during thermal degradation and delayed the onset of decomposition, owing to strong interfacial bonding with the epoxy matrix [70]. Similarly, Gargol et al. demonstrated that glass fiber reinforcement positively influenced the decomposition temperature of hemp-based composites [71]. These results align closely with the present study, where the glass fiber-reinforced F4G2 and H4G2 specimens exhibited lower thermal mass loss and higher decomposition temperatures than their non-hybrid counterparts, highlighting the critical role of glass fibers in improving the thermal performance of natural fiber composites.

**Figure 9:**
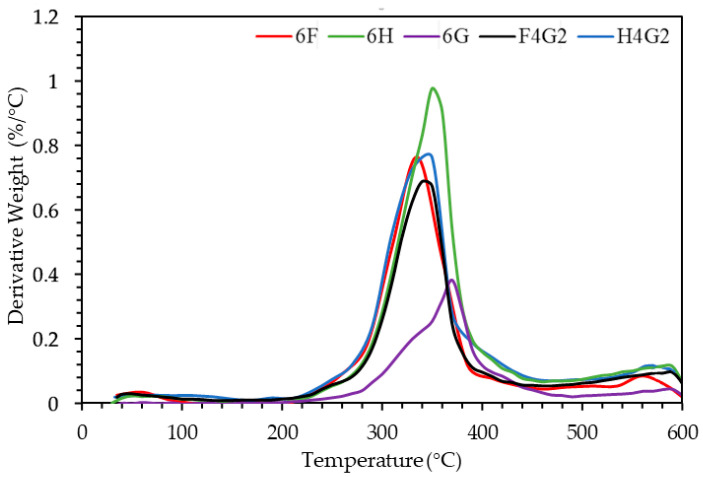
Derivative weight curves of the composites as a function of temperature.

In this study, derivative thermogravimetric (DTG) analysis was conducted to systematically examine the temperature-dependent thermal degradation behaviors of several composite specimens (Figure 9). The findings revealed that specimen 6H, displaying a peak derivative weight loss of 0.98%/°C, undergoes a more rapid thermal degradation process than the other polymer composites evaluated. By contrast, 6F exhibited a peak value of 0.79%/°C and presented a broader degradation temperature range encompassing both lower and higher temperatures than the hemp-containing specimens. Meanwhile, specimen 6G, which initiated degradation at higher temperatures with a peak value of 0.38%/°C, demonstrated enhanced thermal stability relative to the other samples.

Furthermore, in the hybrid composites, including waste glass fibers notably reduced the degradation rate: the F4G2 specimen exhibited a peak derivative weight loss of 0.69%/°C, and the H4G2 specimen exhibited a peak value of 0.77%/°C. Similar DTG behaviors have been reported in the literature. Gargol et al. observed that hemp fiber composites without glass fiber showed a DTG peak at 322.7 °C, with a sharp mass loss of 51.2%. In contrast, hybrid systems exhibited lower degradation rates and improved thermal stability [71]. Likewise, Neto et al. reported peak degradation between 290 and 380 °C for natural fiber composites, with DTG maxima often exceeding 1.0%/°C [30]. Compared to the previous study [30], the lower DTG peak intensities of F4G2 and H4G2 confirm the stabilizing effect of glass fibers, limiting volatile release and slowing matrix decomposition.

### 3.3. DMA of the Composites

**Figure 10:**
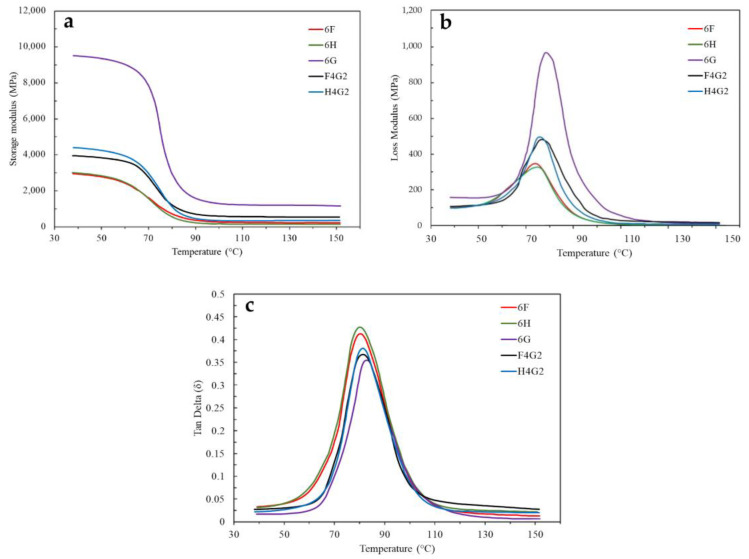
Storage modulus, loss modulus (δ), and tan delta (δ) curves as a function of temperature for all composites. (**a**) Storage modulus curves; (**b**) loss modulus curves (δ); (**c**) tan delta curves (δ).

The storage modulus (E′) is a measure of the energy stored in the elastic structure of a material, corresponding to the material’s elastic response. This variable is particularly useful for evaluating the stiffness and elastic behavior of composites [72]. The variation in storage modulus (E′) as a function of temperature for glass fiber-reinforced and natural fiber-reinforced composites is graphically presented in Figure 10a. The first phase, which lies below the glass transition region and can be referred to as the glassy region, is characterized by restricted polymer chain mobility due to the tightly packed and rigid molecular arrangement. Consequently, a higher storage modulus is observed [73]. The highest storage modulus was recorded in the 6G composite specimen, where the maximum E′ value at 40 °C was approximately 9535.1 MPa. In contrast, at the same temperature, the E′ value of the H4G2 composite was around 4471.7 MPa, followed by the F4G2 composite at approximately 3932.5 MPa. In the case of composites containing waste glass fibers, it has been observed that hybridization with natural fibers accompanied by a reduction in the storage modulus exerts a detrimental effect on the elasticity of the composites. The elevated storage modulus of the 6G composite indicates that the glass fiber predominantly contributes to the enhanced elastic properties of the composite. During the second phase, known as the glass transition region, the disruption of the tightly packed molecular arrangement leads to increased molecular mobility within polymer chains, resulting in a substantial decrease in the storage modulus [73]. A decreasing trend in the storage modulus was observed throughout the 40–90 °C temperature range, with a particularly notable drop occurring between 60 °C and 90 °C. At temperatures exceeding 100 °C, a decrease in the storage modulus values of all composites has been observed, indicating that the matrix transitions into a rubbery state [74].

Unlike the storage modulus (E′), the loss modulus (E″) is considered a fundamental parameter for assessing the viscous behavior of polymeric materials [75]. The variation in the loss modulus with temperature for epoxy matrix composites, ranging from 40 to 150 °C, is presented in Figure 10b. All the composites exhibit a similar trend, displaying a characteristic behavior consisting of three distinct regions. These regions can be defined as follows: a gradual increase in the loss modulus at low temperatures (<70 °C), a pronounced maximum peak at the transition region, and a rapid decrease in the loss modulus at high temperatures. Notably, the 6G composite reaches the highest loss modulus value of 967.84 MPa at approximately 78.46 °C. In contrast, the 6H and 6F composites show relatively lower peak values of 326.97 MPa and 346.59 MPa at lower temperatures, respectively. The hybrid composites (F4G2 and H4G2) achieve maximum loss modulus values of 492.09 MPa and 480.65 MPa, which are intermediate between the pure waste glass fiber and entirely natural fiber composites. These distinct peak points demonstrate the temperature-dependent changes in the composites’ viscous behavior. Moreover, the width of the curve observed in the transition region can be considered a significant indicator of the differences in the composites’ viscous transition behavior and polymer matrix–fiber interface interactions.

Tan delta (δ) is a parameter used to evaluate the viscoelastic properties of polymer composites and represents the ratio of the loss modulus to the storage modulus. Damping properties in polymer materials are related to the balance between the elastic and viscous phases [76]. The variation in tan δ with temperature is shown in Figure 10c. The maximum damping point is considered to be the glass transition temperature (Tg). Tg represents the temperature at which the polymer transitions from the glassy phase to the rubbery phase and is a critical parameter for understanding the thermal and mechanical performance of composites. Specifically, a high glass transition temperature indicates that the material can maintain its rigidity even at high temperatures, demonstrating better thermal stability.

Incorporating glass fibers into natural fiber-reinforced composites has often been reported to increase both storage and loss moduli while reducing tan δ values. This behavior is typically attributed to improved fiber-matrix interfacial bonding and restricted polymer chain mobility, resulting in stiffer and more elastic composite structures [29,30,77]. The experimental results obtained in this study are consistent with these trends. The F4G2 and H4G2 specimens exhibited higher storage and loss moduli and lower tan δ values than the 6F and 6H composites. These findings provide further evidence that integrating glass fibers can effectively enhance the dynamic mechanical behavior of natural fiber-reinforced epoxy composites.

The composites studied in this work exhibited varying glass transition temperatures, depending on both fiber content and layer configuration. Notably, the 6G composite, reinforced with 100% waste glass fibers, showed the highest glass transition temperature of 83.51 °C, indicating that synthetic fibers form a strong interface interaction with the polymer matrix, restricting chain mobility and increasing the glass transition temperature. In contrast, composites made with natural fibers exhibited relatively lower glass transition temperatures, which can be attributed to the weaker interaction between the matrix and these fibers due to their hydrophilic nature. The hybrid configurations showed transition behaviors between the entirely waste glass fiber and fully natural fiber composites, balancing the properties of both fiber types.

In the literature, the damping properties of composites reinforced with glass and natural fibers have been studied, and it has been observed that there is an inverse relationship between the materials’ load-bearing capacities and their tan δ values, with tan δ values decreasing as load-bearing capacity increases [78]. The tan δ values of the polymer composites examined in this study are presented in Table 7. According to the results, the 6G composite exhibited the lowest tan δ value of 0.36, indicating that it possesses a high load-bearing capacity and, consequently, better mechanical performance. In contrast, the tan δ values of the 6F and 6H composites were higher, suggesting that their load-bearing capacities were lower compared to the composites containing synthetic fibers.

### 3.4. Water Absorption Behavior and Aging Test of Composites

**Figure 11:**
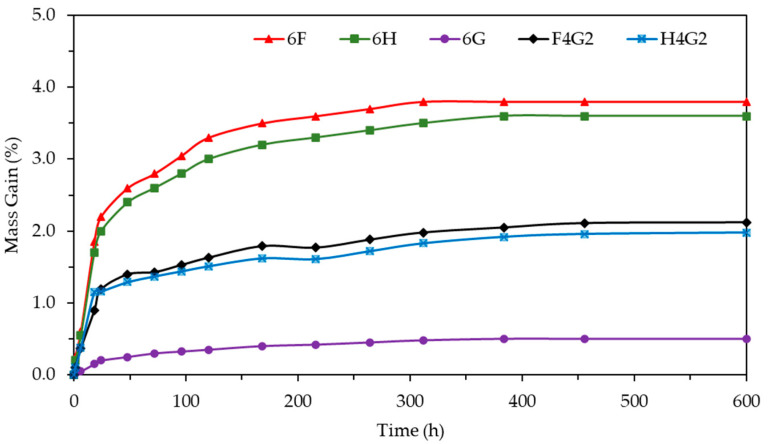
Water absorption graph by the time of the composites.

Experimental results regarding the water absorption behavior of polymer composites are presented in Figure 11, showing the time-dependent percentage of mass increase. The findings indicate that the water absorption rate for all tested samples varies over time. Specifically, in the early stages of the experiment, a rapid increase in mass was observed, especially in natural fiber-reinforced and hybrid composites. This behavior is primarily associated with the hydrophilic properties of natural fibers. Additionally, any porosity and microvoids in the epoxy matrix can facilitate water infiltration into the composite structure, thereby increasing water absorption. The water absorption in natural fiber-reinforced and hybrid composites showed more pronounced changes within the first 50 h. After this period, the rate of mass increase slowed down, approaching the saturation point. The difference in moisture content between the composite material and the environment, as well as the presence of voids/pores on the composite surface, are key factors contributing to this slowdown. The rapid diffusion of water molecules into the laminate in a humid environment and the preferential pathways provided by the voids for water diffusion explain the rapid mass increase in the early stages [79,80]. This process, known as “free water diffusion” in the literature, is kinetically very fast and plays a decisive role during the initial water absorption phase [79]. However, the water infiltrating the composite structure does not lead to significant swelling, as it fills the voids within the material’s mass [81]. Liu et al. reported that incorporating glass fibers into flax-based epoxy composites significantly reduced the water absorption rate, which was attributed to the glass fibers’ barrier effect that hinders moisture ingress [29]. Similarly, Braga and Magalhães observed that jute/glass fiber hybrid composites exhibited lower water absorption than jute-only composites after prolonged immersion, highlighting the positive effect of glass content on moisture resistance [70]. These findings confirm that glass fiber reinforcement, particularly with waste glass fibers, effectively reduces moisture penetration in natural fiber-based composites.

**Figure 12:**
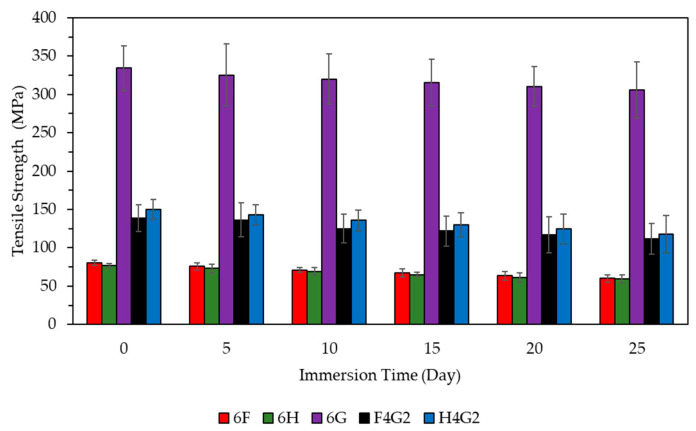
Tensile strength of the 6F, 6H, 6G, F4G2, and H4G2 in distilled water.

In this study, hemp, flax, glass, and hybrid fiber-reinforced composites were subjected to aging conditions by immersion in distilled water for up to 25 days, and their tensile strengths were evaluated. The results are presented in Figure 12.

According to the experimental results, the 6H composite exhibited a tensile strength loss of approximately 22%, while the 6F composite showed a loss of around 25%. The 6G composite, reinforced with waste glass fibers, showed a more limited aging effect, with a tensile strength reduction of approximately 8.5%. In hybrid composites, the F4G2 and H4G2 composites exhibited tensile strength losses of 19% and 21%, respectively. The increased standard deviation values indicate that water absorption and micro-degradation at the fiber-matrix interface were effective to varying degrees. Natural fibers typically have a high moisture absorption capacity, which can cause fiber swelling and loss of dimensional stability. Additionally, swollen fibers may reduce the mechanical properties of the composites and deteriorate the fiber/matrix adhesion [82]. On the other hand, although glass fibers may exhibit physical damage and/or chemical degradation when exposed to water [83], they are more resistant to water aging than natural fibers [84,85]. The hydrophobic qualities of glass fibers and their impact on the diffusion pathways of water molecules were cited as reasons for the reduced water absorption behavior in hybrid composites. The presence of glass fibers in the composite matrix alters the microstructure by disrupting the continuous capillary channels typically formed by hydrophilic natural fibers. This structural alteration makes the water penetration channel more tortuous, which reduces the effective diffusion rate. Improved interfacial bonding between glass fibers and the polymer matrix reduces water intrusion across the fiber-matrix interface. The tighter and less permeable fiber-matrix connections in these regions slow down the rate of hydrolytic breakdown and hinder water mobility. This combined effect, diffusion path alteration, and interface enhancement, is essential for improving the hybrid composite’s moisture resistance and mechanical retention [86,87,88]. Regarding the epoxy matrix, the hydrolytic degradation process in the polymer is characterized by a decrease in crosslink density and an increase in the network’s hydrophilicity [89]. Moreover, production errors that may occur during fiber preparation before composite manufacturing are believed to contribute to some of the deviations in the results, as the 6G composite reinforced with glass fibers uses waste glass fibers, and the orientation of the fibers significantly impacts the mechanical properties.

## 4. Conclusions

This study investigated the mechanical, thermal, water absorption, and dynamic mechanical properties of polymer composites reinforced with waste glass fibers and natural fibers (flax and hemp) in an epoxy matrix. The results indicate that hybridization effectively enhances both structural performance and environmental sustainability. The major findings can be listed as follows:

(1) The incorporation of waste glass fibers increased the tensile strength by 88% and Young’s modulus by 45% compared to composites containing only natural fibers.

(2) Thermogravimetric analysis revealed that hybrid composites exhibited a higher thermal decomposition temperature (369.98 °C) than composites containing only natural fibers (350.96 °C), demonstrating enhanced thermal resistance. Additionally, hybrid composites retained more solid residue at 600 °C, confirming their superior thermal degradation resistance.

(3) The storage modulus of hybrid composites improved by 30% compared to composites containing only natural fibers, ensuring better stiffness and load resistance. The glass transition temperature (Tg) increased, indicating enhanced thermal and mechanical stability under dynamic loading conditions.

(4) The composites composed only of natural fibers absorbed more moisture, whereas hybrid composites exhibited lower water uptake, minimizing swelling and fiber degradation. Tensile strength degradation after prolonged water immersion was 22% in hemp composites, 25% in flax composites, and only 8.5% in waste glass fiber composites, highlighting the superior durability of hybrid configurations.

(5) Combining waste glass fibers with natural fibers offers a sustainable solution by reducing synthetic fiber dependency and promoting eco-friendly material usage.

The results confirm that the hybrid use of waste glass fibers and natural fiber fabrics provides a sustainable and structurally viable alternative for various applications. Considering their enhanced mechanical, thermal, and moisture resistance properties, future research should focus on optimizing the developed materials to increase the capacity of load-bearing elements or eliminate structural deficiencies for strengthening applications. Moreover, further studies could explore fiber-matrix adhesion enhancements, different stacking sequences, and durability under cyclic loading conditions to expand the usability of these composites in infrastructure and construction industries.

## Figures and Tables

**Table 1:** Properties of reinforcement materials.

Specification	Layered Glass Fiber Waste	Hemp Fabric	Flax Fabric
Mass per unit area (g/m^2^)	200	165	179
Density of fiber (g/cm^3^)	2.54	1.48	1.50
Weaving type	Approximately oriented	Multidirectional	Multidirectional

**Table 2:** Tensile test results of hemp and flax fabrics.

Fabric Type	Direction	Breaking Load (N)	Elongation at Break (%)
Hemp	Warp	804.2 ± 77.7	26.7 ± 1.2
Hemp	Weft	764.6 ± 66.4	23.7 ± 0.8
Flax	Warp	397.8 ± 42.3	23.3 ± 2.8
Flax	Weft	388.0 ± 47.1	23.8 ± 5.0

**Table 3:** Layer configuration.

Composite	Thickness (t) (mm)	Layer Configuration	Natural Fiber Volume (%)	Glass Fiber Volume (%)
F6	1.8 ± 0.07	F + F + F + F + F + F	47.9	-
H6	1.9 ± 0.02	H + H + H + H + H + H	42.7	-
G6	2.2 ± 0.10	G + G + G +G + G + G	-	39.1
F4G2	2.3 ± 0.13	F + F + G + G + F + F	24.6	13.8
H4G2	2.2 ± 0.11	H + H + G + G + H + H	23.7	14.4

G: Waste glass fiber; H: hemp fiber; F: flax fiber.

**Table 4:** Tensile test results of polymer composites.

Sample	Thickness (mm)	Elongation at Break (%)	Young’s Modulus (GPa)	Toughness (N.mm)	Tensile Strength (MPa)
6F	1.8	10.8	2.2	19,897.8	80.7 ± 3.6
6H	1.9	7.9	2.4	15,125.8	76.6 ± 2.9
6G	2.2	4.6	9.4	40,969.5	334.2 ± 28.9
F4G2	2.3	4.8	3.5	21,967.0	149.8 ± 12.9
H4G2	2.2	4.7	3.2	18,963.4	138.6 ± 17.4

**Table 5:** A review of mechanical properties of FRP composites for reinforced concrete strengthening applications.

Fiber	Matrix	Tensile Strength (MPa)	Young’s Modulus (GPa)	Strengthening Performance	Ref.
Jute and Flax	Epoxy	35.2–39.2 for jute FRP 55.9–123.0 for flax FRP	1.42–2.03 for Jute FRP 1.34–5.76 for flax FRP	Cost-efficient strengthening and NFRP matched synthetic FRP performance, showing a strengthening effect close to CFRP.	[55]
Flax	Epoxy	123.0–252.0	5.8–10.5	Cost-effective and sustainable strengthening, NFRP provided comparable performance to synthetic FRP, demonstrating significant flexural stiffness improvement and enhanced load-bearing capacity.	[56]
Sisal	Epoxy and polyester	80–104	Sisal FRP (polyester 3 GPa) Sisal FRP (Epoxy 3.2 GPa)	Natural sisal fiber-reinforced polymer (NFRP) composites are effective in enhancing the flexural strength of reinforced concrete beams.	[57]
Kenaf	Epoxy	44.5–119.6	11.7	The structural behavior of beams reinforced with KFRP was found to be close to that of beams reinforced with CFRP.	[58]
Kenaf and Jute	Epoxy	131 for kenaf FRP 136 for jute FRP	13.2 for kenaf FRP 14.54 for jute FRP	NFRP-reinforced beams were found to increase shear loads by 34–36% and to perform similarly to CFRP by increasing their ductility.	[59]
Hemp	Epoxy	156	6.4	Hemp fiber-reinforced composites increased the shear strength and ductility of reinforced concrete deep beams.	[60]
Low-cost Glass	Epoxy	377 MPa	19	Low-cost and efficient strengthening, Lo-G FRP provided significant shear capacity improvement, outperforming CFRP in energy dissipation and peak load enhancement.	[61]
Jute and Glass	Epoxy	29.6 for jute FRP 124.5 for glass FRP	2.8 for jute FRP 5.8 for glass FRP	GFRP increased the bearing capacity of the beams, JFRP increased the ductility, and hybrid GFRP-JFRP systems balanced both properties.	[62]
Low-cost Glass and Sisal	Epoxy	377.6 for glass FRP 79.4 for sisal FRP	18.7 for glass FRP 13.8 for sisal FRP	The natural sisal fiber and glass fiber-reinforced polymer (LC-GFRP) materials significantly enhanced the shear strength and ductility of reinforced concrete beams, reducing the risk of brittle failure and increasing the load-carrying capacity.	[63]
Glass and Aramid	Epoxy	212–302	10.2–14.2	Hybrid FRP (HFRP) laminates significantly increase the flexural strength of reinforced concrete beams reinforced with HFRP laminates, and additional thickness does not increase the strength much when the optimum thickness is exceeded.	[64]
Jute, Jute, and Glass and Glass	Epoxy	17–24 for jute FRP 175 for glass FRP 59–86 for HFRP	2.2–2.5 for jute FRP 13.6 for glass FRP 2.8–3.8 for HFRP	Effective shear strengthening, jute, and jute–glass hybrid FRP enhanced beam performance, providing significant strength gains, improved crack control, and increased deformation capacity.	[65]

**Table 6:** Thermal properties and ash content of the composites.

Composites	Temperature at 5% Mass Loss (°C)	Maximum Decomposition Temperature (°C)	Ash Content (%)
6F	237.68	332.75	18.48
6H	284.54	350.96	16.20
6G	326.04	369.98	67.34
F4G2	264.64	342.75	35.15
H4G2	286.57	346.15	33.55

**Table 7:** Changes in the composites’ storage modulus, loss modulus, and peak points of tan δ.

Composites	E′ (MPa) at 40 °C	E″ (MPa) at 40 °C	E″ (MPa)	Tg (°C)	Tan δ	Tg (°C)
6F	2923.2	99.4	346.59	73.82	0.41	80.29
6H	2979.3	92.4	326.97	75.04	0.43	80.76
6G	9535.1	162.1	967.84	78.46	0.36	83.51
F4G2	3932.5	98.3	492.09	75.77	0.37	81.29
H4G2	4471.7	89.4	480.65	76.87	0.38	81.42

## Data Availability

The datasets presented in this article are not readily available because the data are part of ongoing studies. Requests to access the datasets should be directed to the corresponding author.
